# A Nationwide Survey of Program Directors on Resident Attrition in Emergency Medicine

**DOI:** 10.5811/westjem.2020.10.48286

**Published:** 2020-12-14

**Authors:** Andrew Mittelman, Madeline Palmer, Julianne Dugas, Jordan A. Spector, Kerry McCabe, Alexander Y. Sheng

**Affiliations:** Boston Medical Center, Department of Emergency Medicine, Boston, Massachusetts

## Abstract

**Introduction:**

Despite the burdens that resident attrition places upon programs and fellow trainees, emergency medicine (EM) as a specialty has only begun to explore the issue. Our primary objectives were to quantify attrition in EM residency programs and elucidate the reasons behind it. Our secondary objectives were to describe demographic characteristics of residents undergoing attrition, personal factors associated with attrition, and methods of resident replacement.

**Methods:**

We conducted a national survey study of all EM program directors (PDs) during the 2018–2019 academic year. PDs were asked to identify all residents who had left their program prior to completion of training within the last four academic years (2015–2016 to 2018–2019), provide relevant demographic information, select perceived reasons for attrition, and report any resident replacements. Frequencies, percentages, proportions, and 95% confidence intervals were obtained for program- and resident-specific demographics. We performed Fisher’s exact tests to compare reasons for attrition between age groups.

**Results:**

Of 217 PDs successfully contacted, 118 completed the questionnaire (response rate of 54%). A third of programs (39 of 118) reported at least one resident attrition. A total of 52 residents underwent attrition. Attrition was most likely to occur prior to completion of two years of training. Gender and underrepresented minority status were not associated with attrition. Older residents were more likely to leave due to academic challenges. The most common reported reason for attrition was to switch specialties. Resident replacement was found in 42% of cases.

**Conclusion:**

One-third of programs were affected by resident attrition. Gender and underrepresented minority status were not associated with attrition.

## INTRODUCTION

Resident attrition is often defined as the premature loss of a resident prior to completion of training.^1^,^2^ Attrition has the potential to negatively impact fellow trainees and program leadership.^3^–^6^ It can harm future recruitment efforts.^3^,^7^ Attrition can even indirectly affect patient care by “reducing services to patients and disrupting continuity of care.”^6^ Despite the impact, there is a paucity of literature in emergency medicine (EM) exploring the reasons behind, and the risk factors for, attrition. The existing research on attrition arises primarily from the surgical literature. Little has changed since Naylor et al highlighted over a decade ago that “predictors of performance and attrition have proved to be elusive.”^7^

The field of EM has just begun to address the scope of this important issue. Brockberg et al showed that a quarter of EM residencies are impacted by resident attrition each year.^1^ Our primary objectives were to quantify resident attrition in EM training programs and elucidate the reasons behind it. Our secondary objectives were to describe demographic characteristics of residents undergoing attrition, personal factors associated with attrition, and avenues of resident replacement.

## METHODS

We performed a survey study of all Accreditation Council for Graduate Medical Education (ACGME)- accredited EM residency programs in the United States during academic year 2018–2019. We defined resident attrition as the permanent departure of a trainee from the residency program prior to graduation. Residents on temporary leave who subsequently returned were excluded. Program directors (PDs) were asked to identify all residents who left their program prior to completion of training within the prior four academic years (2015–2016 to 2018–2019).

We used an iterative process to generate the questionnaire. We initially piloted the questionnaire at our institution with three residents, one EM PD and one former EM PD, as well as three associate/assistant program directors (APDs). After incorporating suggestions, we piloted the revised questionnaire again with eight residents, one EM PD, and three APDs from our institution. The questionnaire was finally piloted with two PDs from different institutions. The final version of the distributed questionnaire is shown in Appendix A.

The questionnaire includes demographic data about the residency program including class size, defined as small (≤6 residents per class), medium (7–12), or large (>12), and length (3 vs 4 years). PDs were asked to identify the characteristics of residents who underwent attrition (years of training completed, marriage status, parental status) and the perceived reasons for why each resident left. Selection of multiple reasons for each incidence was permitted. All of the demographic inquiries that would undergo statistical analysis were decided on a priori. We chose the demographics based on our literature review of attrition analyses in other specialties, particularly those characteristics that are more likely known to PDs in order to maintain accuracy of the results and reduce potential missing data. Additional variables were collected to describe the cohort of residents undergoing attrition.

Using the ACGME database,^8^ we identified 241 ACGME-accredited EM programs and gathered a list of PD emails for each training program. The questionnaire was first distributed via email during October 2018. Two reminders were sent to non-responders, first in November 2018 and again in April 2019 after the completion of interview season. Data collection did not occur during interview season, as PDs were less likely to have time to accurately complete the questionnaire. Completion of the questionnaire was voluntary. All questionnaire responses were anonymous. The study was deemed exempt by our institutional review board.

### Data Analysis

Questionnaire creation and data collection were done using Research Electronic Data Capture (REDCap)^9^ hosted at Boston University, CTSI 1UL1TR001430. Frequencies, percentages, proportions, and 95% confidence intervals (CI) were obtained for relevant program- and resident-specific demographics. We used one-sample proportion tests to compare demographics of the sample to those of residents nationwide and to the expected number of attritions per training year. The expected number of attritions per training year was a weighted average of 33% of residents per PGY at three-year programs and 25% of residents per PGY at four-year programs. A chi-squared test was used to assess the association between program length and attrition, and we used Fisher’s exact tests to compare reasons for leaving between gender and age groups. To decrease the probability of a Type I error, we applied the Bonferroni method^10^ wherein the original alpha level of 0.05 was divided by 32 (our total number of hypothesis tests performed) to obtain the conservative alpha level of significance of 0.0016. *P*-values less than 0.0015 were considered statistically significant. We did all analyses using SAS v9.4 (SAS Institute, Cary, NC).

## RESULTS

Of the 241 PD email addresses identified, 24 did not successfully reach the intended recipient (eg, firewalls, email bouncebacks), yielding a total sample size of 217 programs. Of this cohort, 118 PDs successfully completed the questionnaire, representing 49% (118/241) of EM programs nationwide and a response rate of 54% (118/217) among those successfully contacted. Background information regarding the EM program sample is shown in Table 1. Eighty-seven (73.7%) of the programs in our sample were three-year programs, which mirrors the national proportion of three-year EM programs (75.0%; Z = −0.3189, *P* = 0.7498).^11^

Thirty-nine programs (33.1%) reported at least one instance of attrition during the 40-month window of interest (July 2015–October 2018). Seven programs lost two residents, and three programs lost three residents. Twenty-seven (51.9%) instances of attrition occurred in three-year programs and 25 (48.1%) in four-year programs, reflecting that 31% (27/87) of three-year programs and 81% (25/31) of four-year programs were affected by attrition. We noted a significant association between program length and attrition (χ^2^ = 22.8226, *P* < 0.0001). Of those who underwent attrition after completion of two years of training (n = 7), six (85.7%) were enrolled in four-year programs. Based on our sample’s composition of resident attrition occurring in 27 three-year programs and 25 four-year programs, we would expect to see an average of 29% of residents leave per postgraduate year. In contrast, our results suggest that trainees left disproportionately early in training, as 45 residents (86.5%) left before completing two years (Z = 9.1062, *P* < 0.0001).

A total of 52 residents were identified as having experienced attrition. Their characteristics and avenues of replacement are described in Table 2. Of the 52 residents who experienced attrition, 69% (36/52) were men; this proportion does not differ significantly from the nationwide cohort of EM residents, of which 64% (n = 4758) are men (Z = 0.7858, *P* = 0.4320).^11^ Moreover, the proportion of attritions that occurred among underrepresented minority (URM) residents (0.1765, 95% CI, 0.0934–0.3048) was not significantly different from the proportion of URM EM residents nationwide (0.1903, 95% CI, 0.6952–0.9066; Z = −0.2516, *P* = 0.8014).^11^ Finally, among the medical doctor/doctor of osteopathic medicine (MD/DO) subset of our sample (n = 51, after exclusion of n = 1 international medical graduate [IMG]), the MD attrition percentage did not differ significantly from the national composition (70.6% vs 77.1%, *P* = 0.2676).^11^

Of the 52 residents identified in our study, 45 (86.5% (95% CI, 74.4–93.6)) left prior to completion of two years of training. Twenty-two residents who underwent attrition (42.3%) were subsequently replaced in their respective programs. Replacements were most commonly found with the assistance of the Council of Residency Directors in Emergency Medicine (CORD) Listserv or Society of Academic Emergency Medicine Residency Vacancy Services (SAEM). No replacements were found using the Association of American Medical Colleges, openresidencyposition.com, or residentswap.org. Table 3 depicts the perceived reasons for resident attrition. According to PDs, no residents left due to financial concerns, military commitments, or sequelae from a difficult clinical case.

PDs had the option to select multiple reasons for each incidence of attrition. On average, 1.73 (standard deviation = 0.93) reasons for leaving were identified per resident. The most commonly cited reason for attrition was a desire to change specialty. Academic challenges and professionalism issues combined yielded a similar number of resident attritions. Trainees most commonly switched into internal medicine, anesthesia, and family medicine (Table 3).

**Figure 1:**
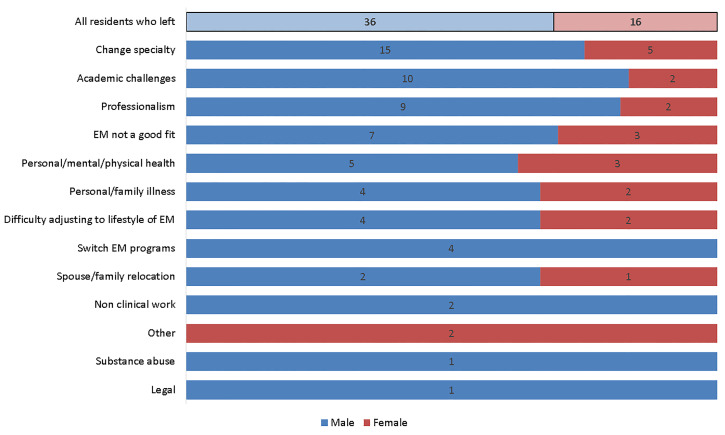
PD perceived reasons for attrition, by gender. *PD*, program director; *EM*, emergency medicine.

The PD-perceived reasons for attrition stratified by gender are shown in Figure 1. Males were relatively more likely than females to leave due to academic challenges (27.8% vs 12.5%) or professionalism (25.0% vs 12.5%), but the differences were not significant (*P* = 0.3010 and *P* = 0.4679, respectively). Substance use and legal troubles were rare. There were no significant associations between gender and any individual reason for leaving.

Residents older than 30 were significantly more likely to leave due to academic challenges (50.0% vs 8.8%, *P* = 0.0015). Relative to younger residents, older residents were *not* more likely to leave due to any other reason including personal/family illness (11.1% vs 11.8%, *P* = 1.0000) or for spouse or family relocation (0.00% vs 8.8%, *P* = 0.5431).

## DISCUSSION

### Program Characteristics

Over the 40-month window of interest, 39 of the 118 EM training programs (33.1%) lost at least one resident prior to training completion. Comparably, Brockberg et al reported that 23% of EM programs experienced attrition each year and more than 80% experienced attrition over the 10-year period of 2007–2016.^1^ Our data is consistent with existing studies demonstrating that while the overall incidence of resident attrition in EM on an individual level is low, a substantial portion of training programs are impacted.

We noted a significant association between program length and attrition. The reason behind the higher rate of attrition in four-year programs is unclear. In-depth qualitative studies are needed to determine whether inherent characteristics of four-year programs foster dissatisfaction resulting in attrition or whether residents simply have more time to leave before completion of training.

### PGY Level

We observed a statistically significant preponderance of attrition occurring prior to completion of two years of training. Although analysis of our data is clouded by the variable length of EM training programs, existing literature in other fields suggests that residents are less likely to experience attrition later in residency.^6^,^12^–^17^

### Age

In our analysis, 54% of EM residents who underwent attrition were 26–30 years old. Nationwide, the median age of an EM resident is 29 years, with 59% being 27–30 years old.^11^ Previous reports on the association of age and attrition are inconsistent. Older age has been previously shown to predict attrition in neurosurgery,^17^ obstetrics and gynecology (OB/GYN),^18^ and general surgery,^7^ while other studies of surgical fields reported no association with age.^4^,^13^,^19^ Naylor et al suggested that age may be predictive of attrition to the extent that “family and lifestyle issues tend to become more important with increasing age.”^7^ This was not observed in our study.

Although older residents were more likely to leave due to perceived academic challenges, they were *not* more likely to leave due to family and lifestyle issues, personal/family illness, or for spouse or family relocation. The differences may be attributable to the fact that that surgical residencies are longer in duration and demand more clinical hours worked compared to EM training, leading to increased opportunity for more lifestyles issues, personal/family illness, and relocation needs to manifest. Our study is the first to report a correlation between age and academic difficulty in any specialty.

### Gender

Among our sample, the proportion of male residents did not differ significantly from that of all EM residents nationwide. Multiple prior studies in the surgical literature suggest that women are more likely to experience attrition than men.^4^,^7^,^12^,^14^,^17^,^19^–^23^ The difference is so large as to imply that “gender has been uniformly associated with an increased risk of attrition in surgical training programs.”^4^ Possible reasons include lack of role models or mentors, discrimination or the perception of it, and sexual harassment.^12^,^20^,^23^ Only two studies (one from plastic surgery and one from OB/GYN) found that men were more likely than women to leave prematurely,^24^,^25^ although findings have been disputed.^6^ A single study in EM evaluating attrition rates between academic years 2006–2007 to 2015–2016 found that women had a higher rate of attrition than men.^2^ We did not observe a gender effect in our study. The discrepancy may be due in part to differences in study methodology, as well as recent efforts to identify and reduce barriers toward becoming a more female-friendly specialty.^26^,^27^

### Underrepresented in Medicine (URM) Status

Of the residents who underwent attrition, 81% were identified by the PD as “not URM.” Existing surgical literature has suggested that race and ethnicity, specifically Hispanic ethnicity, may be predictors of attrition,^21^ while White race and non-Hispanic ethnicity were shown to be “consistently protective” against attrition.^21^ Similarly in OB/GYN, URM status (defined as Black, Native Hawaiian/Pacific Islander, or American Indian/Alaskan Native) has been identified as an independent predictor of attrition.^18^ We did not observe any such effect in our cohort. The proportion of URM attritions did not differ significantly from the proportion of URM EM residents nationwide.^10^

### Marital Status and Children

Of the 52 residents who experienced attrition, 21 were married (40.4%), 27 were unmarried (52%), and in four instances the marriage status was not known to the PD (7.7%). Some studies in the surgical literature have shown marriage to be protective against attrition,^21^,^22^,^24^,^28^,^29^ although others show no association.^4^,^13^ In our dataset, the majority of residents who underwent attrition (74.5%) entered residency without having children, and did not have a new child during their training (82.4%). The existing literature does not report any association between childrearing before or during residency and attrition.^13^,^29^ In fact, one group noted childrearing to be protective against attrition in orthopedic residencies.^22^

### Geographic Factors

The majority (29/52, 55.8%) of residents who underwent attrition had no ties to the geographic area of their residency. A subset grew up in the area (six residents, 11.5%) or had family living in the area (three residents, 5.9%). Prior research suggests a paradoxical impact of having geographic ties and family nearby. In the surgical literature, non-White women with family nearby had attrition rates as high as 39%. Similar trends were noted in men. Males at large surgical programs in the Northeast with family close by were found to have attrition rates as high as 40% – the highest subgroup incidence noted in any male group in the literature.^21^ The authors posited that nearby family may distract trainees from clinical duties.^21^ Ottenhausen et al also observed higher attrition rates in residents training near where they grew up.^19^ Our dataset was too small to identify any association between geographic ties, proximate family, and attrition.

### Medical Training

Thirty-six of the residents who underwent attrition (69.2%) were MD graduates from USA/Canadian allopathic medical schools. By comparison, MDs comprise 77% of the nationwide cohort of EM residents.^11^ Among the MD/DO subset of our data (n = 51, after exclusion of the one IMG), the MD attrition percentage did not differ significantly from the national composition.^11^ Our findings are in agreement with one OB/GYN study that noted similar rates of attrition based upon degree (3.4% for US-trained MDs vs 4.1% for US-trained DOs), although it is worth noting that current trends in MD/DO enrollment are not known in all fields.^25^

### Rank List

Within our cohort, five of the residents (9.6%) who underwent attrition were considered to have been in the top 10% of their programs’ rank lists while four residents (7.7%) were initially ranked in the lower 1/3 of candidates. Since programs match fewer residents in the top 10% (due to the competitive nature of their applications resulting in multiple programs vying to match them) and the bottom third of their rank list (due to lack of interest from the program), fewer are susceptible to attrition. Those considered to be in the top 1/3 and middle 1/3 of their programs’ rank list were the most likely to undergo attrition, and at similar rates (Table 2), likely due to the fact that the majority of matched residents in a program were ranked as such. Without knowing the true denominator of how many residents were ranked at each program, we could not establish an association between rank list position and attrition. Nevertheless, our findings align with existing literature, which suggests that an individual’s position on the rank list is not associated with future attrition.^7^

### Resident Replacement

It is unclear from our data how often programs that experienced resident attrition actually sought replacement. However, the fact that a resident replacement was secured in almost half of the cases illustrates the importance of the process to programs and PDs alike. Unfortunately, the existing literature offers little guidance in finding replacements aside from the reported time and effort it requires.^30^ The majority of resident replacements in our dataset were found using the CORD listserv or SAEM Residency Vacancy Services while several known resources were not used at all. In terms of predicting future performance, there is evidence in the general surgery literature to suggest that replacement residents are just as likely to succeed as those recruited initially in the match.^30^,^31^ The performance and graduation rate of the 22 replacement residents is not known.

### Reasons for Departure

Most instances of attrition were attributed to multiple perceived reasons (mean 1.73), suggesting that attrition is multifactorial. The most common reason for attrition was a desire to change specialty, corroborating findings noted by Lu et al.^2^ Prior research has shown that men and women depart residency for different reasons.^5^ In the surgical specialties, men are more likely to leave for another specialty, while women are more likely to leave due to issues pertaining to their family or spouse (e.g., relocation).^6^,^28^ As shown in Figure 1, our data revealed that males were relatively more likely than females to leave due to academic challenges or professionalism (27.8% vs 12.5% and 25.0% vs 12.5%, respectively), but these differences were not statistically significant. To this end, Lu et al found that men were more likely than women to be “dismissed” from an EM residency.^2^ This is in contrast to data from other fields that has shown no gender gap in dismissal.^6^,^22^ Substance use and legal troubles were rare in our cohort. No attrition was attributed to financial responsibilities, military commitments, or having been involved in a difficult clinical case / poor patient outcome.

PDs cited academic and professionalism concerns more frequently than lifestyle challenges as having contributed to attrition. This is in contrast to studies in the surgical field, where residents more often leave due to lifestyle factors rather than academic performance.^4^,^12^ It is possible that lifestyle issues are less prominent in EM and hence other causes of attrition predominate.

## LIMITATIONS

Our study has several limitations. Our methods did not capture responses from all 241 programs, as 24 recruitment emails “bounced back” and an additional 46% (99/217) were delivered without any response. Nevertheless, our sample is representative of the national cohort as three-year programs comprised 74% of our residencies who responded, nearly identical to the national composition trend (75% of all ACGME-accredited EM training programs are three years in duration).^18^ A source of potential selection bias exists as PDs affected by attrition may have been more or less willing to complete the questionnaire. For example, PDs from programs with few or no recent cases of resident attrition may have been less motivated to complete the questionnaire.

The responses were subject to recall bias, and in several instances the information was unknown to the PDs. The PDs’ responses may have been an inaccurate reflection of the reason(s) for attrition. Additionally, a subset of the respondent programs might have recently received accreditation and had not trained a full cycle of residents. The questionnaire was distributed approximately halfway through academic year 2018–2019, and it is possible that some programs went on to experience attrition after data collection had finished. Furthermore, the endpoint of the window of interest was dynamic due to the competing priorities of interview season. Some PDs accounted for attrition through October 2018 while others did not fill out the questionnaire until April 2019. Although we sought to identify all instances of attrition, we did not specifically ask PDs to identify whether each instance was voluntary or involuntary.

## CONCLUSION

One-third of residencies in this study were affected by resident attrition across the window of interest. Residents who underwent attrition were unlikely to have completed two years. We found no statistically significant difference in attrition among gender in our cohort. Underrepresented minority residents were not more likely to undergo attrition. Older residents were not more likely to experience attrition due to family issues, but were more likely to leave training in the face of academic challenges. Substance use disorder was rare. Nearly half of the lost residents were replaced, using resources made available by EM national organizations. Further rigorous qualitative research is necessary to better illustrate PD and resident perspectives on the impact of and reasons behind resident attrition.

## Supplementary Information



## Figures and Tables

**Table 1:** Program characteristics and attrition rate (n = 118 programs).

Program characteristics	n	%
Average class size		
Small (≤6)	16	13.6
Medium (7–12)	60	50.9
Large (≥13)	42	35.6
Length of residency		
3 years	87	73.7
4 years	31	26.3
Incidents of attrition		
0 attritions	79	67.0
1 attrition	29	24.6
2 attritions	7	5.9
3 attritions	3	2.5

**Table 2:** Characteristics of residents who underwent attrition (n=52) and their avenues of replacement.

Resident characteristics	n	%
Number of residents missing from each class (by expected PGY status at time of questionnaire)		
PGY-1	10	19.2
PGY-2	15	28.9
PGY-3	19	36.5
PGY-4	8	15.4
Completed years of training		
Less than 1 year	13	25.0
1 year	32	61.5
2 years	7	13.5
Gender		
Male	36	69.2
Female	16	30.8
Estimated age		
<26	6	11.5
26–30	28	53.9
31–35	9	17.3
36–40	6	11.5
>40	3	5.8
Underrepresented minority in medicine		
Yes	9	17.3
No	42	80.8
Unsure	1	1.9
Marriage status		
Married	21	40.4
Unmarried	27	51.9
Unsure	4	7.7
Children before starting residency		
Yes	10	19.2
No	38	73.1
Unsure	3	5.8
Missing	1	1.92
New child or became pregnant during residency		
Yes	6	11.54
No	42	80.77
Unsure	3	5.77
Missing	1	1.92
Medical school education		
MD from US/Canada allopathic medical school	36	69.23
DO from US/Canada osteopathic medical school	15	28.85
International medical graduate	1	1.92
Trained in part or completed residency in another specialty before applying to EM		
Yes	6	11.54
No	45	86.54
Missing	1	1.92
Final rank list position		
Top 10%	5	9.62
Top 1/3	16	30.77
Middle 1/3	19	36.54
Lower 1/3	4	7.69
Unknown	8	15.38
Ties to geographic area		
Grew up in the area	6	11.54
College/medical school, worked in area	7	13.46
Has family living in area	3	5.77
No ties to the area	29	55.77
Unknown	6	11.54
Missing	1	1.92
Resident was replaced		
Yes	22	42.3
Using CORD Listserv	7	31.8
Using SAEM Residency Vacancy Services	8	36.4
Using other means	7	31.8
No	30	57.7

*PGY*, Post-Graduate Year; *MD*, Doctor of Medicine; *DO*, Doctor of Osteopathic Medicine; *EM*, Emergency Medicine; *CORD*, Council of Residency Directors in Emergency Medicine; *SAEM*, Society for Academic Emergency Medicine.

**Table 3:** Program director perceived reasons for resident attrition.

Reason for departure	n	%
Pursue another specialty	20	38.5
Internal medicine	6	35.3
Anesthesia	4	23.5
Family medicine	4	23.5
Obstetrics	1	5.9
Surgery	1	5.9
Psychiatry	1	5.9
Academic challenges	12	23.1
Professionalism issues	11	21.2
EM not a good fit for their skills	10	19.2
Personal, mental or physical health issues	8	15.4
Pursue EM training in another program	8	15.4
Difficulty adjusting to lifestyle of EM	6	11.5
Personal/family illness	6	11.5
Spouse or family relocation	3	5.8
Non-clinical work consulting, research, etc.	2	3.9
Other	2	3.9
Legal concerns	1	1.9
Substance abuse	1	1.9

*EM*, Emergency Medicine.
