# Supplementary material for: A Nationwide Survey of Program Directors on Resident Attrition in Emergency Medicine
Source: West J Emerg Med. 2020 Dec 14;22(1):86–93. doi: 10.5811/westjem.2020.10.48286 (PMC7806332; doi:10.5811/westjem.2020.10.48286)
Supplement: Supplementary file 1 [file wjem-22-86-s001.docx]

**Appendix A**

**What is the average class size of your program?**

[ ] Small (6 or fewer per class)

[ ] Medium (7-12 per class)

[ ] Large (13+ per class)

**What is the length of your residency?**

[ ] 3 years

[ ] 4 years

Please provide the total number of residents who have permanently left your program from your:

**Current PGY-1 Class:**

[ ] 0

[ ] 1

[ ] 2

[ ] 3

[ ] 4

[ ] 5

**Current PGY-2 Class:**

[ ] 0

[ ] 1

[ ] 2

[ ] 3

[ ] 4

[ ] 5

**Current PGY-3 Class:**

[ ] 0

[ ] 1

[ ] 2

[ ] 3

[ ] 4

[ ] 5

**Current PGY-4 Class:**

[ ] 0

[ ] 1

[ ] 2

[ ] 3

[ ] 4

[ ] 5

*--For each identified instance of attrition, the following subset of questions is activated--*

**This previous resident successfully completed how many years of training at your program?**

[ ] Less than 1 year

[ ] 1

[ ] 2

[ ] 3

[ ] More than 3 years

**Gender**

[ ] Male

[ ] Female

[ ] Other

**This resident left due to (can check multiple)**

[ ] Personal/Family Illness

[ ] Spouse or family relocation

[ ] Pursue another specialty

Specialty: _________

[ ] Pursue EM training in another program

[ ] Nonclinical work (consulting, research, etc)

[ ] Military commitment

[ ] Financial concerns

[ ] Legal concerns

[ ] Personally affected by involvement in care of patients(s) resulting in a suboptimal/traumatic case outcome

[ ] Academic challenges

[ ] Professionalism issues

[ ] Substance abuse

[ ] Personal, mental, or physical health issues

[ ] EM not a good fit for their skills

[ ] Difficulty adjusting to lifestyle of EM

[ ] Other

Other reason for leaving program: _________

**From your recollection, where did this previous resident reside on your final rank list?**

[ ] Top 10%

[ ] Top 1/3

[ ] Middle 1/3

[ ] Lower 1/3

[ ] I don't remember

**Estimate the age of the previous resident at the time of starting residency**

[ ] < 26 years old

[ ] 26-30 years old

[ ] 31-35 years old

[ ] 36-40 years old

[ ] >40 years old

**Previous resident marriage status**

[ ] Married

[ ] Unmarried

[ ] Unsure

**Did this previous resident have children before starting residency?**

[ ] Yes

[ ] No

[ ] Unsure

**Did this previous resident/their partner have a new child or become pregnant during residency?**

[ ] Yes

[ ] No

[ ] Unsure

**Was this previous resident an under-represented minority in medicine?**

[ ] Yes

[ ] No

[ ] Unsure

**What ties, if any, did this resident have before starting residency to your geographic area? (can check multiple)**

[ ] Grew up in the area

[ ] College/medical school/worked in the area

[ ] Has family living in the area

[ ] No ties to the area

[ ] Unknown

**Previous resident medical school education**

[ ] MD from USA/Canada allopathic medical school

[ ] DO from USA/Canada osteopathic medical school

[ ] International medical graduate

[ ] Other

Other Education: _________

[ ] Unsure

**Has this previous resident been trained (in part or completed residency) in a specialty other than EM before applying to EM residency?**

[ ] Yes

What specialty? _________

[ ] No

**Did you recruit a resident outside the match to replace this previous resident?**

[ ] Yes

How did you find the replacement?

[ ] CORD Listserve

[ ] SAEM

[ ] AAMC

[ ] openresidencypositions.com

[ ] residentswap.org

[ ] Other

Other: _________

[ ] No

*--After accounting for each instance of attrition, the survey ends with a final question--*

**Would you be interested in further conversation regarding your experience with resident attrition and how it has affected your residency program?**

[ ] Yes

Please enter your email address: _________

[ ] No
